# Associations between the Epithelial-Mesenchymal Transition Phenotypes of Circulating Tumor Cells and the Clinicopathological Features of Patients with Colorectal Cancer

**DOI:** 10.1155/2017/9474532

**Published:** 2017-12-21

**Authors:** Fengjie Wu, Jun Zhu, Yongjiang Mao, Xiaomei Li, Baoguang Hu, Dianliang Zhang

**Affiliations:** ^1^Department of General Surgery, The Affiliated Hospital of Qingdao University, Qingdao, Shandong 266011, China; ^2^Department of Gastrointestinal Surgery, The Affiliated Hospital of Binzhou Medical University, Binzhou, Shandong 256603, China; ^3^Department of Ultrasound, The 3rd Affiliated Hospital of Sun Yat-sen University, Guangzhou, Guangdong 510630, China; ^4^Centers for Disease Control and Prevention of Binzhou City, Binzhou, Shandong 256603, China; ^5^Center of Colon and Rectum, Qingdao Municipal Hospital, Qingdao, Shandong 266011, China

## Abstract

In this study, we identified CTCs using the previously reported CanPatrol CTC enrichment technique from peripheral blood samples of 126 patients with colorectal cancer (CRC) and found that CTCs could be classified into three subpopulations based on expression of epithelial cell adhesion molecule (EpCAM) (E-CTCs), the mesenchymal cell marker vimentin (M-CTCs), or both EpCAM and vimentin (biphenotypic E/M-CTCs). Circulating tumor microemboli (CTMs) were also identified in peripheral blood samples. Meanwhile, E-CTCs, M-CTCs, E/M-CTCs, and CTMs were detected in 76.98%, 42.06%, 56.35%, and 36.51% of the 126 patients, respectively. Interestingly, the presence of CTMs and each CTC subpopulation was significantly associated with blood lymphocyte counts and tumor-node-metastasis stage (*P* < 0.001). Lymphocyte counts and the neutrophil-to-lymphocyte ratio (NLR) in patients lacking CTCs were significantly different from those in patients testing positive for CTMs and each CTC subpopulation (*P* < 0.001). Our results indicate that tumor metastasis is more significantly associated with the presence of CTMs and M-CTCs than with other CTC subpopulations and suggest that EMT may be involved in CTC evasion of lymphocyte-mediated clearance.

## 1. Introduction

Colorectal cancer (CRC) is one of the most common types of tumor in industrialized countries. The primary cause of all cancer-related deaths worldwide is metastasis to distant organs. Circulating tumor cells (CTCs), which can be collected by liquid biopsy, are considered to be responsible for metastasis, and they have been widely studied over the past 5 years as potential prognostic markers for various malignancies, including CRC [1–5]. In addition, CTCs are emerging as a possible target for novel anticancer treatments and as markers for monitoring therapeutic efficacy [6–8]. However, distinguishing rare CTCs from the approximate 10^7^ leukocytes/ml and 5x10^9^ erythrocytes/ml in the blood is technically challenging [9]. In early studies, CTCs were detected via the expression of epithelial-specific markers, such as epithelial cell adhesion molecule (EpCAM) and cytokeratins, by immunocytochemistry or reverse transcription-polymerase chain reaction analysis. Since then, markers such as the estrogen receptor, human epidermal growth factor receptor 2, and immune-checkpoint genes have also been used to facilitate the detection of CTCs in peripheral blood.

Epithelial-to-mesenchymal transition (EMT) and the concomitant acquisition of invasive potential play an important role in the biological progression of metastasis. Recent work has suggested that EMT phenotypes in CTCs may be responsible for metastasis, raising interest in the correlation between EMT-CTC subpopulations and features of metastatic cancer [10–13]. The ability to phenotype CTCs using EMT markers may be helpful for identifying the most aggressive CTC subpopulations and determining an appropriate treatment approach. A recent study described a novel technique for the identification and classification of CTCs into three subpopulations based on the expression of epithelial (E-CTC), biphenotypic epithelial/mesenchymal (E/M-CTC), and mesenchymal (M-CTC) markers [14].

In the present study, we identified CTCs based on EMT phenotypes using the previously reported CanPatrol CTC enrichment technique from peripheral blood samples of patients with CRC and investigated the clinical significance of CTCs with different EMT phenotypes in CRC by examining the relationships between clinicopathological parameters and the relative abundance of three circulating EMT-CTC subpopulations.

## 2. Materials and Methods

### 2.1. Patients and Sample Collection

This study examined blood samples collected from 126 patients who were diagnosed with CRC between July 2014 and June 2016 at the Affiliated Hospital of Binzhou Medical University (Binzhou, China). All cancer diagnoses were confirmed by histopathological analysis. The study protocol and patient consent forms were approved by the Ethics Review Committee of the Affiliated Hospital of Binzhou Medical University, and all patients signed the consent forms before inclusion in the study. The first 2 ml peripheral blood collected was discarded to avoid potential skin cell contamination from the venipuncture, and 7.5 ml blood was collected into a 10 ml tube containing 2.5 ml of EDTA anticoagulant. All blood samples were processed within 4 h of collection. Patients did not undergo surgery or received any other treatment prior to sample collection.

To avoid bias, the physicians and research scientists who collected samples, collected and/or analyzed data, or evaluated the CTC subtypes were blinded to the patient clinical characteristics. A CTC count of >2/7.5 ml blood was considered to be a CTC-positive result.

### 2.2. Detection of CTCs

CTCs were identified using the previously reported CanPatrol CTC enrichment technique [15], which is a two-step method involving filter-based isolation of CTCs followed by detection of EMT markers (EpCAM and vimentin) using an RNA in situ hybridization (ISH) method. Briefly, erythrocytes were removed from blood samples via the addition of erythrocyte lysis buffer, and leukocytes were removed using a size-based filtration system with an 8 *μ*m pore filter membrane. CTC subpopulations were then identified via differential expression of EpCAM, vimentin, and the leukocyte marker receptor-type tyrosine-protein phosphatase C (CD45) using RNA ISH, as described below. Cells were stained with 4′,6-diamidino-2-phenylindole (DAPI) for 5 min and analyzed with a fluorescence microscope (Olympus Corporation, Tokyo, Japan) using a ×100 objective. CTCs were identified as DAPI^+^ CD45^−^ cells with the one of the following phenotypes: EpCAM^+^ vimentin^−^ (E-CTCs), EpCAM^+^ vimentin^+^ (biphenotypic E/M-CTCs), or EpCAM^−^ vimentin^+^ (M-CTCs).

### 2.3. RNA ISH

RNA ISH, a technique based on branched DNA (bDNA) signal amplification, was performed as described by Wu et al. [14]. The captured probe sequences for CD45, vimentin, and EpCAM and the sequences for the bDNA signal amplification probes were as previously described [14] and were obtained from Invitrogen (Thermo Fisher Scientific Inc., Waltham, MA, USA). Briefly, enriched cells from the CanPatrol technique were treated with a protease (Qiagen GmbH, Hilden, Germany) and hybridized with captured probes specific for EpCAM, vimentin, and CD45 for 2 h at 42°C. Unbound probes were removed by washing three times with a wash buffer. Cells were incubated with preamplifier solution at 42°C for 20 min, cooled, washed three times with 1 ml wash buffer, and incubated with amplifier solution for 1 h at room temperature. Fluorescently labeled probes (Alexa Fluor 594 for vimentin, Alexa Fluor 488 for EpCAM, and Alexa Fluor 647 for CD45) were added, and the cells were incubated at 42°C for 20 min. Finally, the cells were washed, stained with DAPI at room temperature for 5 min, and analyzed by fluorescence microscopy.

### 2.4. Statistical Analysis

Statistical analysis was performed using SPSS 21.0 (IBM Corp., Armonk, NY, USA) and GraphPad Prism 6.0 (GraphPad Software Inc., La Jolla, CA, USA) software. Associations between CTCs and clinical parameters were evaluated using a two-sided *χ*^2^ test or one-way analysis of variance. *P* < 0.05 was considered to indicate a statistically significant difference, and all tests were two-sided.

## 3. Results

### 3.1. Detection of CTCs in the Peripheral Blood of Patients with CRC

**Figure 1:**
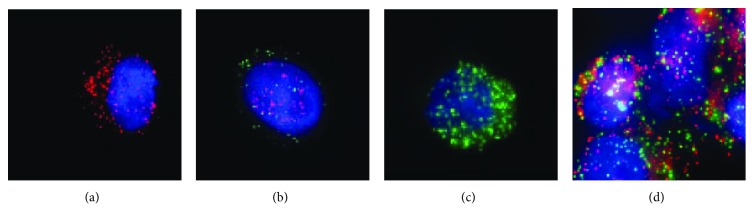
Detection of CTCs and CTMs in the peripheral blood of patients with colorectal cancer. (a) EpCAM expression (red fluorescence) in epithelial CTCs (E-CTCs). (b) EpCAM and vimentin expression (red and green fluorescence, resp.) in epithelial/mesenchymal CTCs (E/M-CTCs). (c) Vimentin expression (green fluorescence) in mesenchymal CTCs (M-CTCs). (d) CTMs, consisting of E/M-CTCs. Magnification, ×100. CTC, circulating tumor cell; CTMs, circulating tumor microemboli; EpCAM, epithelial cell adhesion molecule.

CTCs were identified according to differential expression of the epithelial marker EpCAM, the mesenchymal marker vimentin, and the leukocyte marker CD45, following enrichment of peripheral blood cells using a filter-based method. The cells were classified as E-CTCs, E/M-CTCs, and M-CTCs. Circulating tumor microemboli (CTMs) were also detected in the peripheral blood samples (Figure 1).

### 3.2. Patient Characteristics and Detection of CTCs

The clinicopathological characteristics of the 126 patients with CRC included in this study are summarized in Table 1. E-CTCs were detected in the peripheral blood of 97 patients (76.98%), while E/M-CTCs, M-CTCs, and CTMs were detected in 56.35%, 42.06%, and 36.51% of patients, respectively. Notably, patient samples that were E-CTC positive were not necessarily positive for E/M-CTCs, M-CTCs, or CTMs, whereas all CTM-positive samples were also positive for E-CTCs, E/M-CTCs, or M-CTCs.

### 3.3. Correlation of CTCs with Clinicopathological Features

Among the clinicopathological features analyzed, lymph node metastasis and tumor-node-metastasis (TNM) stage were significantly associated with the presence of CTCs. As shown in Table 2, CTCs were present in significantly more patients with lymph node metastasis than in those without (*P* = 0.0001), and the presence of CTCs was positively correlated with TNM stage (*P* = 0.0001, *r* = 0.45). However, other factors, including age, sex, and tumor differentiation degree, exhibited no correlation with CTC count. We also compared the frequency of CTC-positive and CTC-negative patients following categorization into carcinoembryonic antigen-positive and CA 19-9-positive groups, based on reference values of 3.5 ng/ml and 27 U/l, respectively. However, there were no significant differences in the proportions of CTC-positive and CTC-negative patients in these subgroups.

**Figure 2:**
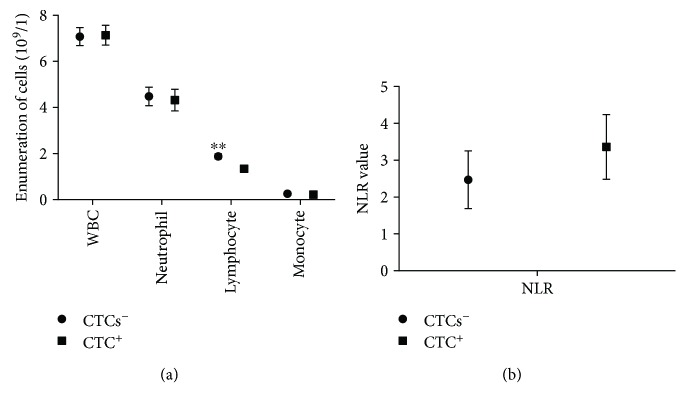
Immune cells in the peripheral blood of patients with colorectal cancer. (a) Difference in WBC, neutrophil, lymphocyte, and monocyte counts in CTC-positive and CTC-negative patients. (b) Neutrophil-to-lymphocyte ratio in CTC-positive and CTC-negative patients. ^∗∗^*P* < 0.01. WBC, white blood cell; CTC, circulating tumor cell.

We also quantified the immune cells in the peripheral blood of patients with CRC and analyzed the associations between immune cell parameters and the presence of CTCs (Figure 2). There were no significant differences between the CTC-negative and CTC-positive patient groups with respect to total white blood cell (WBC), neutrophil or monocyte counts, or the neutrophil-to-lymphocyte ratio (NLR). However, there was a significant difference in lymphocyte count between the CTC-positive and CTC-negative patient groups (*P* = 0.0001).

### 3.4. Associations between CTC-EMT Subpopulations and Clinicopathological Features

**Figure 3:**
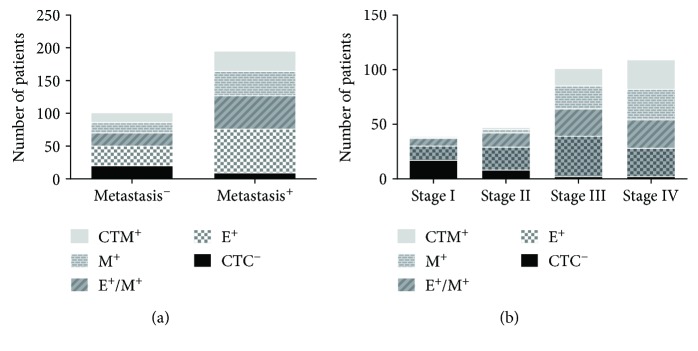
Distribution of CTC subpopulations in patients with CRC. (a) Distribution of CTC subpopulations in patients with or without metastasis. (b) Distribution of CTC subpopulations in patients at different stages of CRC. CTC, circulating tumor cell; CRC, colorectal cancer.

The associations between the presence of CTC subpopulations of each EMT phenotype and clinicopathological features were analyzed. As shown in Figure 3, E-CTCs were detected in all patients regardless of the presence or absence of lymph node metastases; however, M-CTCs and CTMs were detected in significantly more patients with lymph node metastasis than without. With respect to the TNM stage, CTMs were fewer in patients with stage I and II disease, whereas M-CTCs were most common in patients with stage I CRC. In addition, all patients with stage IV disease were positive for all three subtypes of CTC.

**Figure 4:**
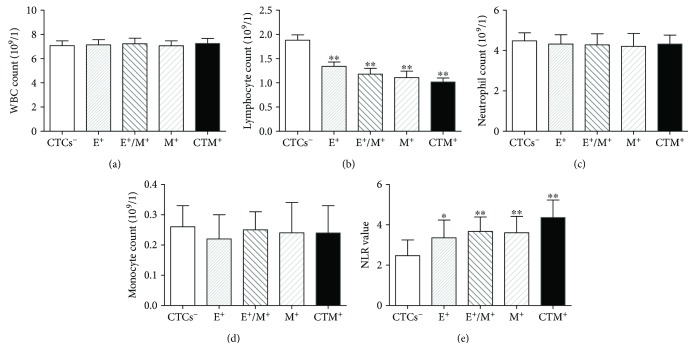
Quantification of WBCs in patients with colorectal cancer. (a) Total WBC, (b) lymphocytes, (c) neutrophils, (d) monocytes, and the NLR in patients lacking CTCs or positive for E-CTCs, M-CTCs, or E/M-CTCs. ^∗^*P* < 0.05 and ^∗∗^*P* < 0.01. WBC, white blood cell; NLR, neutrophil-to-lymphocyte ratio; CTC, circulating tumor cell; E, epithelial; M, mesenchymal.

We also analyzed the association between each CTC subpopulation and the WBC count. As shown in Figure 4, patients testing positive for any subpopulation of CTC had significantly lower lymphocyte counts and lower NLRs than patients testing negative for that subpopulation (*P* < 0.001).

## 4. Discussion

CTCs derived from epithelial tumors often show highly heterogeneous expression or complete loss of epithelial markers, hampering the detection of CTCs by conventional methods based on antibody-mediated capture or cytokeratin staining [16, 17]. Moreover, recent studies have reported that phenotypic alterations, such as the overexpression of mesenchymal markers and the loss of epithelial markers, are common in CTCs in general [18, 19], suggesting a need for CTC detection methods that include biomarkers unaffected by the EMT process. In this study, we isolated CTCs from patients with CRC using the cell size- and phenotype-based CanPatrol technique, which has been reported to detect CTCs with a high efficiency [14]. Using this method, we detected CTCs with different EMT phenotypes in 97 out of 126 (76.98%) patients with CRC. Furthermore, it was discovered that M-CTCs and CTMs were most common in patients with advanced cancer, whereas the CTMs were strongly positive for mesenchymal markers and were absent from the samples containing predominantly E-CTCs from patients with early-stage cancer. These results support the notion of a role for EMT in tumor metastasis and indicated that the role of M-CTCs and CTMs might be more important that the other subpopulations in terms of the risk of disease progression. We then analyzed the association between CTC subtypes and patient clinical characteristics. Notably, there was a significant association between each subpopulation and both lymph node metastasis and TNM stage. These characteristics were more strongly associated with M-CTCs and CTMs than with E-CTCs and E/M-CTCs. This implies that M-CTCs and CTMs might play more important roles than the other subpopulations in tumor metastasis. Unlike other reports involving CTC detection and evaluation of the clinical significance of CTCs, we investigated the role of CTCs as well as the clinical significance of each EMT phenotype in tumor metastasis.

Although millions of tumor cells are shed from a primary tumor into the bloodstream during metastasis, only a few cells survive to form new lesions [20]. Chen et al. [21] posited that the number of CTCs may be influenced not only by the type of primary tumor but also by the number of immune cells in the blood. To investigate this, we examined the association between CTCs and immune cell counts, including the NLR. We observed that the lymphocyte count and NLR were significantly increased in the CTC-negative patient group compared with the positive group. Moreover, there was a negative correlation between CTCs and lymphocytes and a positive correlation between CTCs and NLR. These results suggested that lymphocytes may play a major role in the clearance of circulating CTCs, which is consistent with the findings reported by Chen et al. [21]. In the present study, we also found that E-CTCs and CTMs were negatively correlated with lymphocyte counts and that the patients who were positive for E-CTCs and CTMs had significantly lower circulating lymphocyte counts than did the CTC-negative group or the group lacking E-CTCs. Recently, a review [22] of the mechanisms of immune surveillance and evasion of CTCs reported that CTCs might shed or otherwise restrict the presentation of ligands involved in recognition by natural killer cells or cytotoxic T lymphocytes or downregulate the expression of other factors that promote activation of tumor-specific immune responses. Based on our results and the above review, we therefore hypothesized that CTCs might shed certain immune-associated molecules via EMT to escape from immune cell-mediated clearance.

Nevertheless, there are some limitations to the present study. First, additional CTC detection methods should be investigated to determine whether our results were influenced by the detection method. Secondly, the number of patients in our study was small and the results should be interpreted with caution, and the role of the EMT in CTC immune evasion should be confirmed by experiments *in vitro*.

In conclusion, our results indicate that the M-CTC subpopulation or CTMs might play more important roles than other CTC subpopulations in tumor metastasis. In addition, EMT may be involved in CTC avoidance of lymphocyte-mediated clearance.

## Figures and Tables

**Table 1:** Clinical characteristics of the patients and detection of CTCs.

Parameter	Number of patients	Percentage (%)
Total	126	
*Gender*		
Male	68	53.97
Female	58	46.03
*Age*		
<60	60	47.62
>60	66	52.38
*Differentiation*		
Poor	27	21.43
Well and moderate	99	78.57
*Lymph node metastasis*		
Yes	70	55.56
No	56	44.44
*TNM stage*		
I	17	13.49
II	39	30.95
III	41	32.54
IV	26	20.63
*CEA*		
≤3.5 ng/ml	45	35.71
>3.5 ng/ml	81	64.29
*CA199*		
≤27 U/ml	76	60.32
>27 U/ml	50	39.68
*CTCs*		
Negative	29	23.02
E	97	76.98
E/M	71	56.35
M	53	42.06
CTM	46	36.51

**Table 2:** Correlation between CTC detection rate and clinical characteristics.

Parameter	Number of patients with CTCs		*P* value	*R*	*χ* ^2^
	Negative	Positive			
*Gender*			0.684	—	0.17
Male	15	46			
Female	14	51			
*Age*			0.779	—	0.08
≤60	12	43			
>60	17	54			
*Differentiation*			0.319	—	1.00
Poor	11	47			
Well and moderate	18	50			
*Lymph node metastasis*			0.0001	0.33	13.50
No	20	30			
Yes	9	67			
*TNM stage*			0.0001	0.45	30.54
I	17	13			
II	8	21			
III	2	37			
IV	2	26			
*CEA*			0.05	0.18	3.848
≤3.5 ng/ml	16	72			
>3.5 ng/ml	13	25			
*CA199*			0.108	—	2.59
≤27 U/ml	14	31			
>27 U/ml	15	66			
